# 2-Chloro-4-fluoro-*N*-phenyl­benzamide

**DOI:** 10.1107/S1600536809024787

**Published:** 2009-07-04

**Authors:** Zhengde Tan, Yi Bing, Shen Fang, Zhao Kai, Yang Yan

**Affiliations:** a College of Chemistry and Chemical Engineering, Hunan Institute of Engineering, Xiangtan 411104, People’s Republic of China; b Guangxi Institute of Standards and Technology, Nanning 530022, People’s Republic of China

## Abstract

In the title compound, C_13_H_9_ClFNO, the dihedral angle between the two aromatic rings is 13.6 (2)°. In the crystal, the mol­ecules are linked by inter­molecular N—H⋯O hydrogen bonds into chains extending along the *c*-axis direction.

## Related literature

For the chemical and pharmacological properties of amides, see: Arrizabalaga *et al.* (1984 ▶); Šladowska *et al.* (1999 ▶).
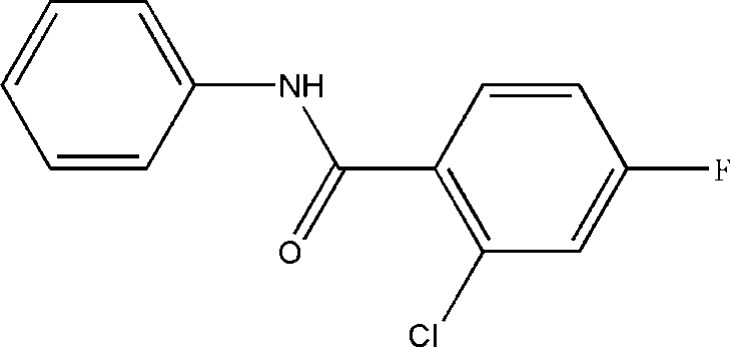


## Experimental

### 

#### Crystal data

C_13_H_9_ClFNO
*M* *_r_* = 249.66
Monoclinic, 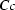

*a* = 22.262 (3) Å
*b* = 5.6452 (6) Å
*c* = 9.6743 (12) Å
β = 105.832 (2)°
*V* = 1169.7 (2) Å^3^
*Z* = 4
Mo *K*α radiation
μ = 0.32 mm^−1^
*T* = 298 K
0.45 × 0.40 × 0.27 mm

#### Data collection

Bruker SMART CCD diffractometer
Absorption correction: multi-scan (*SADABS*; Sheldrick, 1996 ▶) *T*_min_ = 0.869, *T*_max_ = 0.919
2887 measured reflections
1671 independent reflections
1470 reflections with *I* > 2σ(*I*)
*R*_int_ = 0.023

#### Refinement

*R*[*F* ^2^ > 2σ(*F* ^2^)] = 0.032
*wR*(*F* ^2^) = 0.079
*S* = 1.04
1671 reflections
154 parameters
2 restraints
H-atom parameters constrained
Δρ_max_ = 0.21 e Å^−3^
Δρ_min_ = −0.12 e Å^−3^
Absolute structure: Flack, (1983 ▶), 637 Friedel pairs
Flack parameter: 0.04 (7)

### 

Data collection: *SMART* (Siemens, 1996 ▶); cell refinement: *SAINT* (Siemens, 1996 ▶); data reduction: *SAINT*; program(s) used to solve structure: *SHELXS97* (Sheldrick, 2008 ▶); program(s) used to refine structure: *SHELXL97* (Sheldrick, 2008 ▶); molecular graphics: *SHELXTL* (Sheldrick, 2008 ▶); software used to prepare material for publication: *SHELXTL*.

## Supplementary Material

Crystal structure: contains datablocks I, global. DOI: 10.1107/S1600536809024787/si2182sup1.cif

Structure factors: contains datablocks I. DOI: 10.1107/S1600536809024787/si2182Isup2.hkl

Additional supplementary materials:  crystallographic information; 3D view; checkCIF report

## Figures and Tables

**Table 1:** Hydrogen-bond geometry (Å, °)

*D*—H⋯*A*	*D*—H	H⋯*A*	*D*⋯*A*	*D*—H⋯*A*
N1—H1⋯O1^i^	0.86	2.04	2.857 (3)	159

Symmetry code: (i) 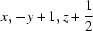.

**Fig. 1.**
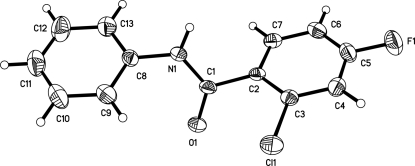
The molecular structure of the title compound showing 50% probability displacement ellipsoids for non-H atoms.

**Fig. 2.**
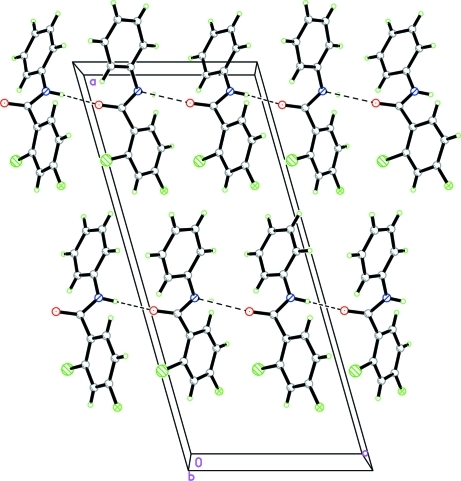
Crystal packing of the title compound, showing the hydrogen bonds as dashed lines.

## supplementary crystallographic information

### Comment

The chemical and pharmacological properties of acid amides have been investigated extensively, owing to their chelating ability with metal ions and to their potentially beneficial chemical and biological activties (Arrizabalaga *et al.*, 1984; Šladowska *et al.*, 1999). As part of our studies on the synthesis and characterization of related compounds, we report here the synthesis and crystal structure of 2-chloro-4-fluoro-*N*-phenylbenzamide. The C=O bond length is 1.224 (3) Å, indicating that the molecule is in the keto form (Fig. 1). In the crystal structure, the molecules are linked by intermolecular N—H···O hydrogen bonds into chains, which extend along the *c* direction (Table 1 and Fig. 2)

### Experimental

A solution of 2-chloro-4-fluorobenzoyl chloride (10 mmol) in 50 ml toluene was added to a solution of aniline (10 mmol) in 10 ml toluene. The reaction mixture was refluxed for 1 h with stirring then the resulting white precipitate was obtained by filtration, washed several times with ethanol and dried *in vacuo* (yield 90%). Elemental analysis calculated: C, 62.54; H, 3.63; N, 5.61; found: C, 62.51; H, 3.62; N, 5.59. Crystals were obtained by slow evaporation of a solution in methanol after one week.

### Refinement

H atoms were placed geometrically and refined using a riding model, with C—H = 0.93Å and N—H = 0.86 Å, respectively. *U*_iso_(H) = 1.2*U*_eq_(C, N).

### Crystal data

**Table:**

C_13_H_9_ClFNO	*F*(000) = 512
*M**_r_* = 249.66	*D*_x_ = 1.418 Mg m^−^^3^
Monoclinic, *C**c*	Mo *K*α radiation, λ = 0.71073 Å
*a* = 22.262 (3) Å	Cell parameters from 1638 reflections
*b* = 5.6452 (6) Å	θ = 2.9–27.0°
*c* = 9.6743 (12) Å	µ = 0.32 mm^−^^1^
β = 105.832 (2)°	*T* = 298 K
*V* = 1169.7 (2) Å^3^	Block, colorless
*Z* = 4	0.45 × 0.40 × 0.27 mm

### Data collection

**Table:**

Bruker SMART CCD diffractometer	1671 independent reflections
Radiation source: fine-focus sealed tube	1470 reflections with *I* > 2σ(*I*)
graphite	*R*_int_ = 0.023
φ and ω scans	θ_max_ = 25.0°, θ_min_ = 1.9°
Absorption correction: multi-scan (*SADABS*; Sheldrick, 1996)	*h* = −24→26
*T*_min_ = 0.869, *T*_max_ = 0.919	*k* = −6→5
2887 measured reflections	*l* = −11→11

### Refinement

**Table:**

Refinement on *F*^2^	Secondary atom site location: difference Fourier map
Least-squares matrix: full	Hydrogen site location: inferred from neighbouring sites
*R*[*F*^2^ > 2σ(*F*^2^)] = 0.032	H-atom parameters constrained
*wR*(*F*^2^) = 0.079	*w* = 1/[σ^2^(*F*_o_^2^) + (0.0432*P*)^2^ + 0.1416*P*] where *P* = (*F*_o_^2^ + 2*F*_c_^2^)/3
*S* = 1.04	(Δ/σ)_max_ < 0.001
1671 reflections	Δρ_max_ = 0.21 e Å^−^^3^
154 parameters	Δρ_min_ = −0.12 e Å^−^^3^
2 restraints	Absolute structure: Flack, (1983), 637 Friedel pairs
Primary atom site location: structure-invariant direct methods	Flack parameter: 0.04 (7)

### Special details

**Table:**

Geometry. All e.s.d.'s (except the e.s.d. in the dihedral angle between two l.s. planes) are estimated using the full covariance matrix. The cell e.s.d.'s are taken into account individually in the estimation of e.s.d.'s in distances, angles and torsion angles; correlations between e.s.d.'s in cell parameters are only used when they are defined by crystal symmetry. An approximate (isotropic) treatment of cell e.s.d.'s is used for estimating e.s.d.'s involving l.s. planes.
Refinement. Refinement of *F*^2^ against ALL reflections. The weighted *R*-factor *wR* and goodness of fit *S* are based on *F*^2^, conventional *R*-factors *R* are based on *F*, with *F* set to zero for negative *F*^2^. The threshold expression of *F*^2^ > σ(*F*^2^) is used only for calculating *R*-factors(gt) *etc*. and is not relevant to the choice of reflections for refinement. *R*-factors based on *F*^2^ are statistically about twice as large as those based on *F*, and *R*- factors based on ALL data will be even larger.

### Fractional atomic coordinates and isotropic or equivalent isotropic displacement parameters (Å^2^)

**Table:**

	*x*	*y*	*z*	*U*_iso_*/*U*_eq_	
Cl1	0.25827 (4)	0.57325 (11)	0.03581 (7)	0.0567 (2)	
F1	0.19086 (10)	−0.1122 (4)	0.2865 (2)	0.0839 (7)	
N1	0.42846 (11)	0.5122 (4)	0.3097 (2)	0.0439 (5)	
H1	0.4200	0.4944	0.3907	0.053*	
O1	0.39721 (10)	0.4168 (4)	0.0742 (2)	0.0619 (6)	
C1	0.38996 (13)	0.4115 (4)	0.1951 (3)	0.0407 (6)	
C2	0.33604 (12)	0.2803 (4)	0.2241 (2)	0.0362 (6)	
C3	0.27498 (13)	0.3337 (4)	0.1537 (3)	0.0392 (6)	
C4	0.22559 (14)	0.2060 (5)	0.1747 (3)	0.0479 (7)	
H4	0.1845	0.2458	0.1281	0.057*	
C5	0.23956 (15)	0.0173 (6)	0.2675 (3)	0.0538 (8)	
C6	0.29854 (16)	−0.0470 (5)	0.3395 (3)	0.0513 (8)	
H6	0.3061	−0.1772	0.4008	0.062*	
C7	0.34669 (13)	0.0887 (5)	0.3182 (3)	0.0457 (7)	
H7	0.3875	0.0510	0.3681	0.055*	
C8	0.48249 (13)	0.6470 (5)	0.3095 (3)	0.0428 (7)	
C9	0.47880 (18)	0.8278 (6)	0.2111 (4)	0.0632 (8)	
H9	0.4415	0.8593	0.1422	0.076*	
C10	0.5315 (2)	0.9607 (7)	0.2168 (5)	0.0802 (12)	
H10	0.5296	1.0810	0.1501	0.096*	
C11	0.5862 (2)	0.9185 (6)	0.3187 (5)	0.0805 (12)	
H11	0.6212	1.0107	0.3220	0.097*	
C12	0.58956 (18)	0.7403 (7)	0.4161 (5)	0.0810 (10)	
H12	0.6269	0.7102	0.4855	0.097*	
C13	0.53723 (17)	0.6047 (6)	0.4110 (4)	0.0637 (9)	
H13	0.5395	0.4838	0.4775	0.076*	

### Atomic displacement parameters (Å^2^)

**Table:**

	*U*^11^	*U*^22^	*U*^33^	*U*^12^	*U*^13^	*U*^23^
Cl1	0.0651 (5)	0.0510 (4)	0.0528 (5)	0.0117 (4)	0.0138 (3)	0.0092 (4)
F1	0.0792 (15)	0.0871 (14)	0.0939 (17)	−0.0334 (11)	0.0379 (13)	0.0012 (12)
N1	0.0456 (13)	0.0597 (13)	0.0304 (13)	−0.0075 (11)	0.0170 (10)	−0.0029 (10)
O1	0.0661 (15)	0.0934 (16)	0.0314 (11)	−0.0216 (11)	0.0221 (10)	−0.0078 (10)
C1	0.0450 (15)	0.0521 (15)	0.0278 (15)	0.0011 (13)	0.0145 (12)	0.0014 (12)
C2	0.0434 (15)	0.0422 (14)	0.0235 (13)	−0.0018 (12)	0.0101 (11)	−0.0035 (10)
C3	0.0473 (16)	0.0376 (13)	0.0340 (15)	0.0002 (11)	0.0133 (13)	−0.0045 (11)
C4	0.0416 (16)	0.0548 (16)	0.0473 (17)	−0.0005 (13)	0.0122 (13)	−0.0097 (14)
C5	0.060 (2)	0.0530 (17)	0.056 (2)	−0.0156 (18)	0.0290 (18)	−0.0052 (14)
C6	0.070 (2)	0.0450 (17)	0.0436 (18)	−0.0034 (14)	0.0226 (16)	0.0029 (13)
C7	0.0496 (18)	0.0528 (16)	0.0337 (15)	0.0032 (13)	0.0097 (14)	−0.0016 (13)
C8	0.0431 (18)	0.0492 (15)	0.0410 (16)	−0.0041 (13)	0.0199 (14)	−0.0049 (12)
C9	0.065 (2)	0.0698 (19)	0.057 (2)	−0.0049 (17)	0.0210 (17)	0.0119 (17)
C10	0.088 (3)	0.072 (2)	0.090 (3)	−0.018 (2)	0.042 (3)	0.014 (2)
C11	0.062 (3)	0.084 (3)	0.102 (3)	−0.021 (2)	0.034 (3)	−0.005 (2)
C12	0.048 (2)	0.088 (3)	0.103 (3)	−0.0086 (18)	0.0121 (18)	0.002 (2)
C13	0.056 (2)	0.071 (2)	0.062 (2)	−0.0065 (17)	0.0130 (17)	0.0067 (16)

### Geometric parameters (Å, °)

**Table:**

Cl1—C3	1.743 (3)	C6—H6	0.9300
F1—C5	1.361 (4)	C7—H7	0.9300
N1—C1	1.330 (4)	C8—C13	1.362 (5)
N1—C8	1.424 (3)	C8—C9	1.382 (4)
N1—H1	0.8600	C9—C10	1.381 (5)
O1—C1	1.224 (3)	C9—H9	0.9300
C1—C2	1.500 (4)	C10—C11	1.363 (6)
C2—C3	1.377 (4)	C10—H10	0.9300
C2—C7	1.392 (4)	C11—C12	1.366 (6)
C3—C4	1.375 (4)	C11—H11	0.9300
C4—C5	1.373 (4)	C12—C13	1.383 (5)
C4—H4	0.9300	C12—H12	0.9300
C5—C6	1.357 (5)	C13—H13	0.9300
C6—C7	1.378 (4)		
			
C1—N1—C8	125.4 (2)	C6—C7—C2	122.0 (3)
C1—N1—H1	117.3	C6—C7—H7	119.0
C8—N1—H1	117.3	C2—C7—H7	119.0
O1—C1—N1	124.3 (3)	C13—C8—C9	120.0 (3)
O1—C1—C2	120.8 (2)	C13—C8—N1	119.7 (3)
N1—C1—C2	114.9 (2)	C9—C8—N1	120.3 (3)
C3—C2—C7	117.5 (2)	C10—C9—C8	118.9 (3)
C3—C2—C1	122.1 (2)	C10—C9—H9	120.5
C7—C2—C1	120.2 (2)	C8—C9—H9	120.5
C4—C3—C2	122.2 (2)	C11—C10—C9	121.1 (3)
C4—C3—Cl1	117.8 (2)	C11—C10—H10	119.5
C2—C3—Cl1	119.92 (19)	C9—C10—H10	119.5
C5—C4—C3	117.1 (3)	C10—C11—C12	119.8 (3)
C5—C4—H4	121.5	C10—C11—H11	120.1
C3—C4—H4	121.5	C12—C11—H11	120.1
C6—C5—F1	118.8 (3)	C11—C12—C13	119.8 (4)
C6—C5—C4	123.9 (3)	C11—C12—H12	120.1
F1—C5—C4	117.3 (3)	C13—C12—H12	120.1
C5—C6—C7	117.2 (3)	C8—C13—C12	120.5 (3)
C5—C6—H6	121.4	C8—C13—H13	119.7
C7—C6—H6	121.4	C12—C13—H13	119.7
			
C8—N1—C1—O1	2.1 (4)	C4—C5—C6—C7	0.7 (4)
C8—N1—C1—C2	−179.5 (2)	C5—C6—C7—C2	−1.6 (4)
O1—C1—C2—C3	−58.5 (3)	C3—C2—C7—C6	1.1 (4)
N1—C1—C2—C3	123.1 (3)	C1—C2—C7—C6	−175.2 (2)
O1—C1—C2—C7	117.5 (3)	C1—N1—C8—C13	−133.3 (3)
N1—C1—C2—C7	−60.9 (3)	C1—N1—C8—C9	49.8 (4)
C7—C2—C3—C4	0.5 (4)	C13—C8—C9—C10	0.9 (5)
C1—C2—C3—C4	176.6 (2)	N1—C8—C9—C10	177.8 (3)
C7—C2—C3—Cl1	179.33 (19)	C8—C9—C10—C11	−1.0 (6)
C1—C2—C3—Cl1	−4.5 (3)	C9—C10—C11—C12	0.8 (6)
C2—C3—C4—C5	−1.4 (4)	C10—C11—C12—C13	−0.4 (6)
Cl1—C3—C4—C5	179.8 (2)	C9—C8—C13—C12	−0.5 (5)
C3—C4—C5—C6	0.7 (4)	N1—C8—C13—C12	−177.4 (3)
C3—C4—C5—F1	−178.8 (2)	C11—C12—C13—C8	0.2 (6)
F1—C5—C6—C7	−179.8 (3)		

### Hydrogen-bond geometry (Å, °)

**Table:**

*D*—H···*A*	*D*—H	H···*A*	*D*···*A*	*D*—H···*A*
N1—H1···O1^i^	0.86	2.04	2.857 (3)	159

Symmetry codes: (i) *x*, −*y*+1, *z*+1/2.
